# A Case Report of a Metastatic Papillary Carcinoma of the Thyroid

**DOI:** 10.7759/cureus.55627

**Published:** 2024-03-06

**Authors:** Abhilasha Bhargava, Chandrashekhar Mahakalkar, Shivani Kshirsagar, Shivani Bothara, Jayashree Bhawani

**Affiliations:** 1 General Surgery, Datta Meghe Institute of Higher Education and Research, Wardha, IND; 2 Radiodiagnosis, Datta Meghe Institute of Higher Education and Research, Wardha, IND; 3 Pathology, Datta Meghe Institute of Higher Education and Research, Wardha, IND

**Keywords:** goiter, neck tumor, thyroidectomy, thyroid, papillary thyroid carcinoma, metastatic thyroid carcinoma

## Abstract

Neck lumps can be a symptom of thyroid and parathyroid gland metabolic diseases, and papillary thyroid carcinoma is reported in some cases. It is commonly observed in middle-aged people with a female predominance. Papillary carcinoma of the thyroid is the most common type of thyroid cancer, originating from the thyroid gland cells. It is slow-growing and less aggressive, but it has been reported to have the ability to affect nearby lymph nodes and other organs. It is associated with the RET protooncogene, NTRK1, and MET genes. Early detection is crucial, especially for middle-aged patients. Treatment typically involves thyroidectomy and radioactive iodine therapy, with the need for hormone replacement therapy. Fine-needle aspiration cytology (FNAC) is an efficient and cost-effective tool for diagnosing neck swellings, leading to a conclusive diagnosis of the mass. We present a case of a 60-year-old Indian female with swelling over the neck for the past six years, which was recently accompanied by dyspnea, hand tremors, and palpitations. The ignored neck mass was found to be a hyper-echoic mass with macro calcifications and cystic degeneration on ultrasonography, confirmed as papillary thyroid carcinoma by FNAC, followed by a complete thyroidectomy and uneventful follow-up.

## Introduction

Papillary carcinoma of the thyroid gland is a common type of thyroid cancer. It originates in the thyroid gland follicular cells. The thyroid gland is responsible for the production of hormones, which control various bodily functions. Papillary thyroid carcinoma is reported to have characteristic nuclear features such as multifactorial etiology [1]. Nuclear features such as the presence of "Orphan Annie eye nuclei," multiple nucleoli, enlarged nuclei, an irregular nuclear contour, the presence of nuclear grooves, and others. Papillary carcinoma is generally seen as having slow growth and a less aggressive form, which has the ability to spread to nearby lymph nodes and, in some cases, to other organs [2]. Papillary carcinoma of the thyroid has been associated with the RET protooncogene, NTRK1, and MET genes. Most individuals diagnosed with this form of thyroid cancer do have a good prognosis, especially if the cancer is detected early, particularly in patients <45 years old. A fine-needle aspiration biopsy has been found to be cost-effective and aid in early diagnosis [2,3]. Treatment typically involves thyroidectomy and may also include radioactive iodine therapy to eliminate remnant thyroid cells. Hormone replacement therapy is necessitated to regulate the hormonal functions lost due to thyroidectomy [4].

## Case presentation

**Figure 1:**
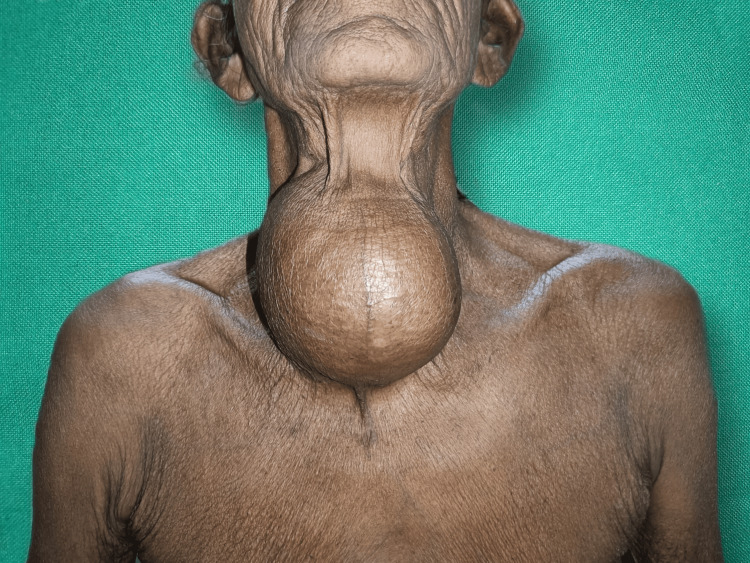
Physical presentation of the anterior aspect of the neck swelling

**Figure 2:**
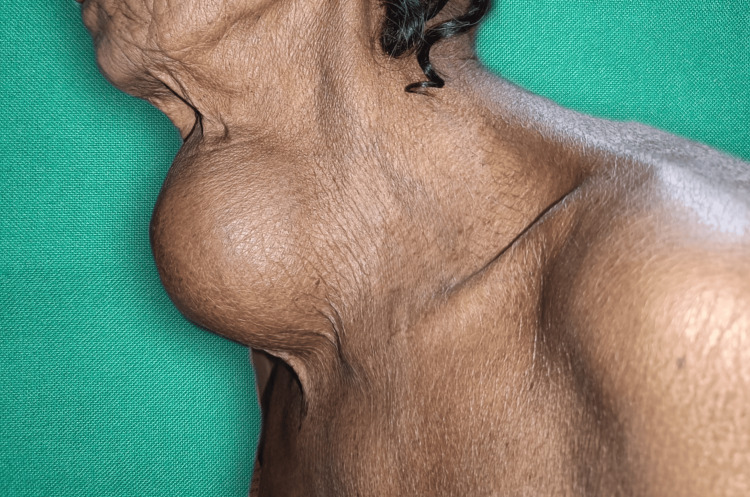
Clinical presentation of the left pane of the neck swelling

**Figure 3:**
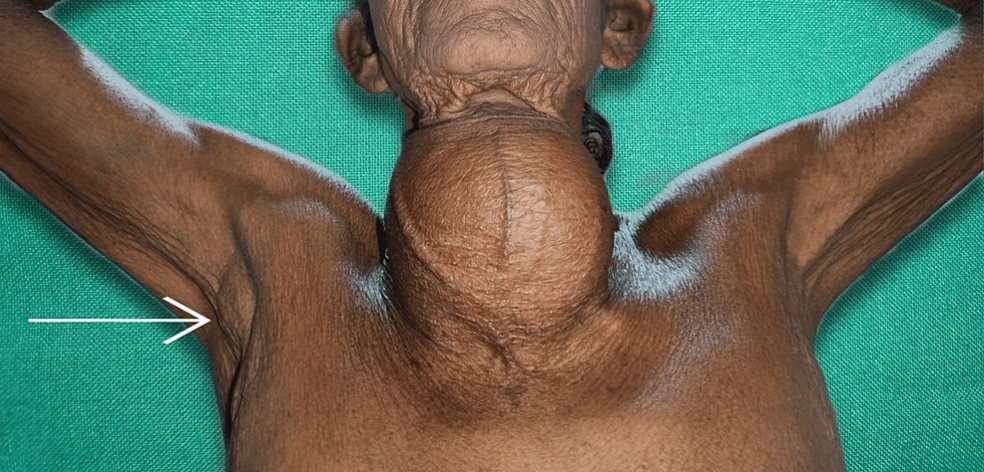
Physical presentation of the neck swelling with an arrow showing the right axillary lymphadenopathy

A 60-year-old Indian female came with complaints of swelling over the anterior aspect of the neck for six years, with an insidious onset and rapid, painless progression of the swelling noticed in the last three months. It was associated with dyspnea, hand tremors, and palpitation. There were no complaints of dysphagia, voice change, or any co-morbidities. Clinically, the swelling was solitary and firm in consistency on the anterior aspect of the neck, measuring 12 x 8 cm in size (Figures 1-3).

**Figure 4:**
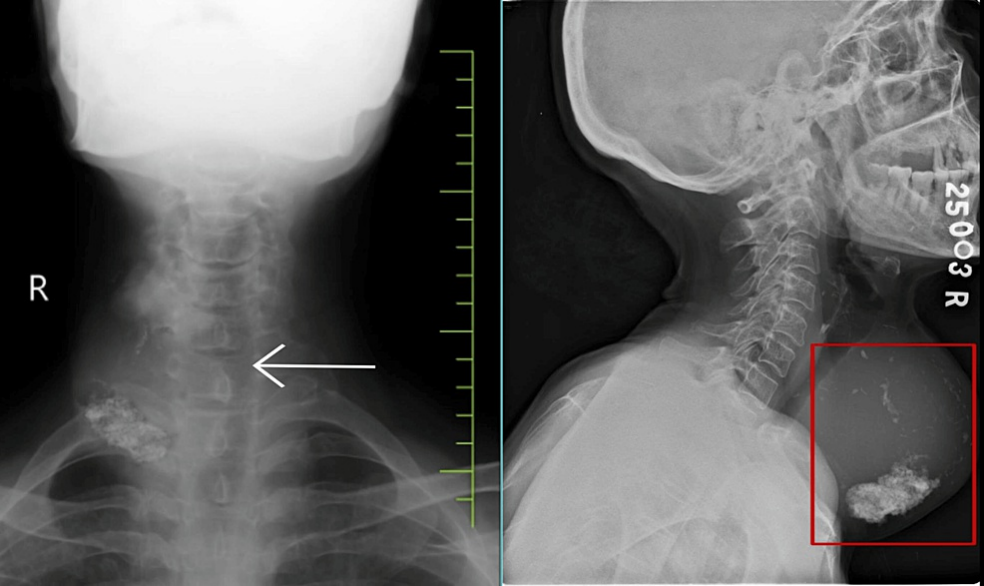
Neck radiograph in anterior-posterior view (left) and lateral view (right) White arrow showing a tracheal shift toward the left side. Neck swelling in the lateral view (red box) with evidence of macro calcification within

The swelling involved both lobes of the thyroid gland and the isthmus, along with evidence of engorged veins. There was no retrosternal extension of the gland. The patient had palpable right axillary lymph nodes in the central and medial groups, the largest measuring 2 x 2 cm. The patient's thyroid profile was suggestive of hyperthyroidism with elevated T3 (4.2 ng/ml) and T4 (13 µg/dl) values and was started on tab carbimazole 10 mg twice a day. The radiograph of the neck showed a well-defined swelling over the anterior aspect of the neck, more toward the right, with significant macro calcifications. There is evidence of a mass effect in the form of slight tracheal compression. On the anterior view, it is suggestive of tracheal deviation toward the left side (Figure 4).

Ultrasonography of the neck revealed a heterogeneously enlarged hyper-echoic mass involving both thyroid lobes and the isthmus, with calcifications and inter-cystic spaces. The lesion was approximately 12 x 10 cm in size with vascularity. No evidence of infiltration over bilateral carotid arteries was noted. There was evidence of significant bilateral reactive lymph nodes in the upper jugulodigastric regions, along with right axillary lymphadenopathy, the largest measuring 2 x 2.2 cm. An ultrasonogram of the neck was suggestive of papillary carcinoma of the thyroid with calcifications and cystic degeneration along with axillary metastasis (Video 1).

**Video 1:** Ultrasonography of the neck

**Figure 5:**
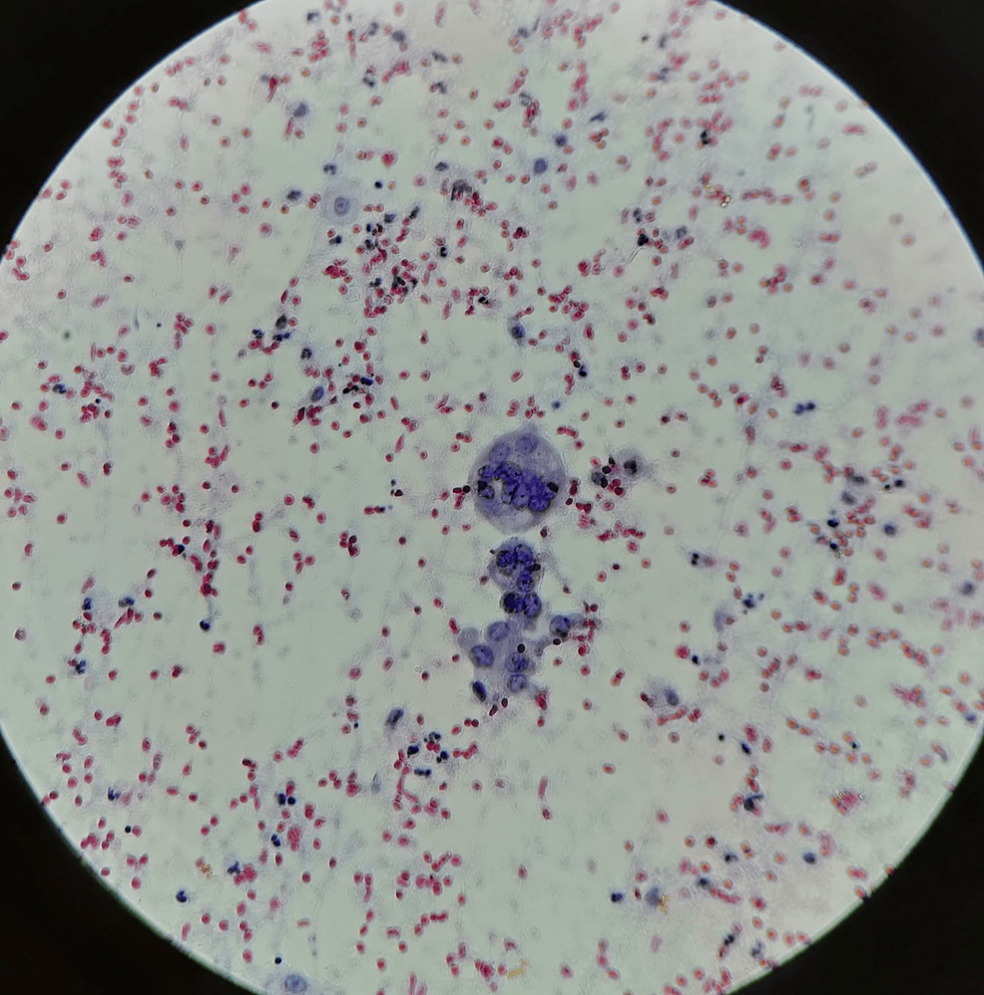
Cytology slide showing evidence of syncytialized giant cells in the tissue

On fine-needle aspiration cytology (FNAC), there was evidence of syncytialized giant cells in the tissue, suggesting a papillary neoplastic etiology of the thyroid gland (Figure 5).

**Figure 6:**
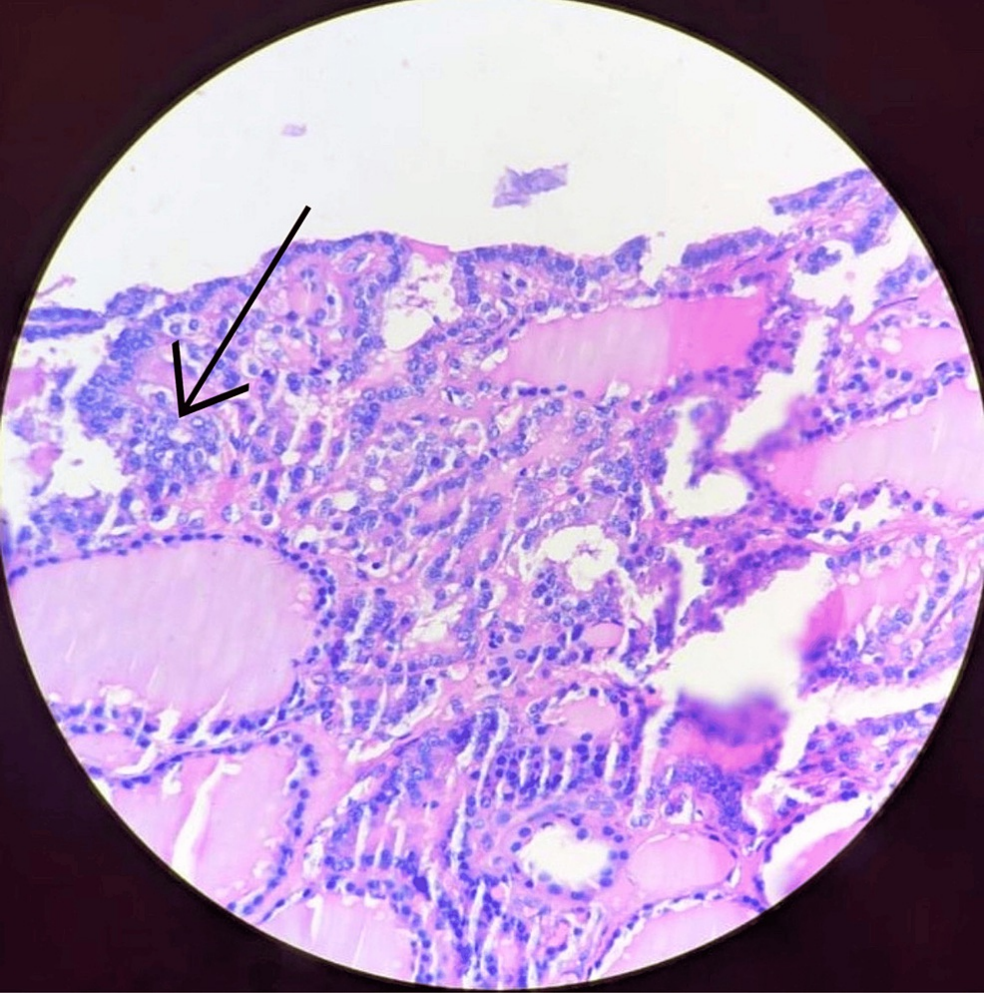
Histopathology slide showing optically clear nuclei or ground glass appearance known as "Orphan Annie eye nuclei" suggestive of a papillary carcinoma of the thyroid Arrow indicating the "Orphan Annie eye nuclei"

After six months, the thyroid profile was repeated, which was suggestive of euthyroidism, and the patient was posted for further management. The patient was subjected to a total thyroidectomy with a modified radical neck dissection and right axillary clearance. The histopathology slide showed evidence of Orphan Annie nuclei in the glandular tissue and intra-nuclear inclusions with tumor cells in resected axillary lymph nodes, confirming the diagnosis of metastatic papillary carcinoma of the thyroid on histopathology (Figure 6).

Postoperatively, tab levothyroxine (100 mcg) was started. At the three-month follow-up, the patient had no fresh complaints, and the scar site was noted to be healthy. The limitation of the case report is the unavailability of the postoperative photograph.

## Discussion

A neck lump may be a symptom of thyroid and parathyroid gland metabolic diseases. Asymptomatic individuals might also develop a clinical presentation in the form of goiter. Thyroid cancer is the most frequent of the endocrine malignancies [5]. Thyroid malignancy is common in females, constitutes nearly 3% of all cancers, and has been reported with a trend of increased incidence in the last few decades [6]. Associated genetic changes reported to be associated with these carcinomas are observed in the anomalous expression of pathways involved in RAS-RAF-MEK-MAP kinase. RAS or BRAF mutations are reported in the activation of MEK1 and MEK2 with a high rate of expression in papillary thyroid carcinoma cells and might be suggestive of aggressive disease forms and poorly differentiated phenotypes [7]. It has been observed as a low-malignant tumor with a good prognosis and timely diagnosis in young individuals. The majority of cases of papillary thyroid carcinoma are observed to occur in middle-aged individuals with a female predominance [2]. The current case presented with an enlarged 12 x 8 cm in the neck, which might be a result of neglected goiter over a period of time. Swelling of the neck might have underlying reasons such as lymphadenopathy and metaplastic carcinomas. A research study on neck swelling reported FNAC as an efficient and cost-effective tool with nearly no risk and minimum complications, leading to a conclusive diagnosis of the superficial mass. The study reported the diagnostic accuracy of FNAC for these cancers, with a few cases as inconclusive [8]. In similarity, the FNAC report of this patient concluded papillary thyroid carcinoma and was further subject to its management. FNAC continues to be a trustworthy investigation for head and neck swellings in the neck and the head.

## Conclusions

Any neck mass progressive in nature should not be ignored, as it might be the result of certain lymphadenopathy or malignancy, such as papillary thyroid carcinoma, which might cause adverse outcomes if not timely addressed. Regular follow-up is important to monitor for any recurrence or spread. Overall, the prognosis of papillary carcinoma is generally favorable, with a good success rate of the treatment and long-term survival.
